# Managing Distrust-Induced Risk with Deposit in Supply Chain Contract Decisions

**DOI:** 10.1155/2014/961394

**Published:** 2014-06-18

**Authors:** Guanghua Han, Ming Dong, Qi Sun

**Affiliations:** ^1^Sino-US Global Logistics Institute, Shanghai Jiao Tong University, Shanghai 200030, China; ^2^Antai College of Economics & Management, Shanghai Jiao Tong University, Shanghai 200052, China; ^3^School of Business Administration, Contemporary Business and Trade Research Center, Zhejiang Gongshang University, Hangzhou 310018, China; ^4^Zheshang Research Center, Zhejiang Gongshang University, Hangzhou 310018, China

## Abstract

This paper studies the trust issue in a two-echelon supply chain information sharing process. In a supply chain, the retailer reports the forecasted demand to the supplier. Traditionally, the supplier's trust in the retailer's reported information is based on the retailer's reputation. However, this paper considers that trust is random and is also affected by the reputation and the demand gap. The supplier and retailer have been shown to have different evaluations regarding the degree of trust. Furthermore, distrust is inherently linked to perceived risk. To mitigate perceived risk, a two-stage decision process with an unpayback deposit contract is proposed. At the first stage, the supplier and the retailer negotiate the deposit contract. At the second stage, a Stackelberg game is used to determine the retailer's reported demand and the supplier's production quantity. We show that the deposits from the retailer's and supplier's perspectives are different. When the retailer's reported demand is equal to the supplier's forecasted demand, the retailer's evaluation of the deposit is more than that of supplier's. When the retailer's reported demand is equal to the retailer's forecasted demand, the deposit from the retailer's perspective is at the lowest level.

## 1. Introduction

In a two-tier supply chain with one retailer and one supplier, the retailer forecasts the market demand and shares the information to the supplier. To maximize its benefit, the information sender often has the intention of covering up the real information and sharing distorted information. Accordingly, trust plays a special role in information sharing and is specified as the information receiver's willingness to rely on the shared information to make decisions. The receiver can update his belief about the information based on his/her level of trust [1]. Thus, the trust level is critical to decision making and it is usually measured by statistics of related indexes.

To the best of our knowledge, trust is usually evaluated only by the reputation of the trustee, which includes past experience, peer recommendation, and so forth [2]. A great deal of research on trust is rooted in this view. Since “trust is a psychological state comprising the intention to accept vulnerability based upon positive expectations of the intentions or behavior of another” [3], the trustee's trust in received information is also affected by current intuition, rather than by the trustee's reputation. In this paper, we consider a supply chain consisting of a supplier (he) and a retailer (she). The supplier relies on the retailer's reported demand information to decide his production quantity. To ensure an abundant supply, the retailer distorts the demand information. In other words, there is a gap between the reported demand information and the supplier's own forecasted demand (demand gap for short). Obviously, the supplier's trust decreases in tandem with the demand gap. Accordingly, in this context, we separate the facts that affect a supplier's trust into two classes: reputation and demand gap. In the electronic online market, the trust level is usually qualified by evaluation in related indexes or by multiple evaluations among transaction partners [4]. Trust is usually considered as a belief or a cognitive stance that is finally quantified by a subjective probability [5, 6]. In this paper, we consider trust as a random variable and follow some distributions.

Perceived risk and risk management are inherently linked to the uncertainty of trust [7]. For example, a customer who usually shops in the traditional way (in a physical store) is reluctant to purchase on the web due to the sense of perceived risk. In the brick-and-mortar retail store, customers can touch and even try the product before making a purchasing decision. This can strengthen his trust in the product and thereby reduce the perceived risk [8]. In the supplier and retailer supply chain, once a supplier relies fully on the reported demand information, he perceives the negative outcomes resulting from uncertain information. Lower trust means higher perceived risk for the decision makers; therefore, risk management approach may need to be used to mitigate the perceive risk. The perceived risk has been found to influence the decision maker's decisions and his profit [9]. Traditionally, risk management technologies, such as system optimization technology [10] and coordination contract [11], are adopted to mitigate the risk. In this study, the supplier's perceived risk is seen as psychological and is directly brought about by distrust on the uncertain demand information. In order to mitigate the supplier's perceived risk, a distrust-based unpayback deposit (deposit in the context for short) contract is employed to mitigate the potential failure. The more the supplier trusts the retailer's reported demand information, the less deposit will be charged. The distrust-induced risk management is applied as a two-stage negotiation contract. At the first stage, the supplier and the retailer negotiate the deposit rule as it pertains to the possible quantity of the reported demand. At the second stage, according to the supplier's potential reaction decision on capacity quantity, the retailer determines the reported demand to maximize his profit. These two-stage negotiation processes are described in numerical studies.

The contents of this paper can be summarized as follows. In Section 1 we consider trust as a random variable consisting of two factors: reputation and demand gap; then a two-stage negotiation is proposed to manage the distrust-induced risk with a deposit. In Section 2, we provide a literature review regarding the relevant research fields.  Section 3 gives the definition of the trust and the trust evaluation model and then proposes a distrust-based deposit rule. In Section 4, we set up a two-stage negotiation contract to show how the supplier uses a trust-based deposit to mitigate the perceived risk. Then Section 5 makes use of some experimental studies to show how the trust-induced risk management contract is implemented in supply chain decisions.

## 2. Literature Review

Trust is a psychological state relying on the integrity, ability, relationship, or character of a person. Some research has been carried out on the connotation of trust [12]. In these papers, the researchers have given different definitions of trust, definitions that consist of one or more of two attributes: (1) trust as a psychological state and (2) trust having the ability to affect a decision maker's decisions.

Since trust is a kind of psychological state, it is not easy for it to be quantified. Multiparticipator involved evaluation systems [13] and reputation-based calculations are used to calculate trust level. Recently, the trust issue has been studied in supply chain information sharing. For example, Ã*Ű*zer et al. [1] explore the role that trust plays in supply chain information sharing and study the corresponding operational decisions considering trust. Ebrahim-Khanjari et al. [14] study operational decisions of a three-tier supply chain considering the information receiver's distrust. These two researchers introduce trust in operation management, and take trust as a tool to filter shared information. Different decision makers might have different evaluations of trust values regarding the same target based on psychological theories. In practice, the trust level is evaluated based on the data surveyed [15] and can be regressed as some distributions. If the supplier fully trusts the reported demand, then his trust level is as large as 1; otherwise, the trust value is equal to 0 if the supplier fully distrusts the reported demand [14]. Reputation is gradually built up, based on past experiences, on a set of criteria [2]; a good reputation usually means a high level of trust [16].

Moreover, trust is also determined by social norms, environment, life experience, the decision makers' personalities, and so on [17]. In this study, the supplier gets demand information from two channels: his own forecast and the retailer's report. When the demand information from these two channels do not match (demand gap), the supplier does not fully trust the demand information from any single channel [18]. Higher information mismatch leads to lower trust level [19]. Thus, we consider the supplier's trust as resulting from two facts: the retailer's reputation and demand gap. Based on this assumption, a new trust evaluation model is set up in this study.

Traditionally, coordination contracts are adopted to mitigate risks [20], and some financial instruments (e.g., deposit, insurance, options, etc.) are also used to enhance the flexibility of the decision system [21]. In such researches, the risk mostly results from the uncertainty of cost [22], demand [23]. Trust is an important factor in risk management, affecting judgments of risk and benefit [24]. However some literature also proves that risk is inherently linked to trust [25]. On the other hand, distrust proportionately increases the decision maker's perceived risk. For example, trust is usually considered as an important factor in the investment decision to reduce corresponding risks [26]. Thus, this paper focuses on the mitigation of distrust which results from perceived risk. First, an approach is provided to quantify the trust level, and a two-stage negotiation process is proposed to mitigate the distrust-induced perceived risk.

In short, in supply chain information sharing, the information receiver usually does not fully trust the information from the reporters when having to make decisions relying on it. This paper suggests an information filter contract based on a trust model and then builds a two-stage negotiation process to mitigate the supplier's risk. The mainly used notations are listed in Table 1.

## 3. Modeling Trust

**Figure 1:**
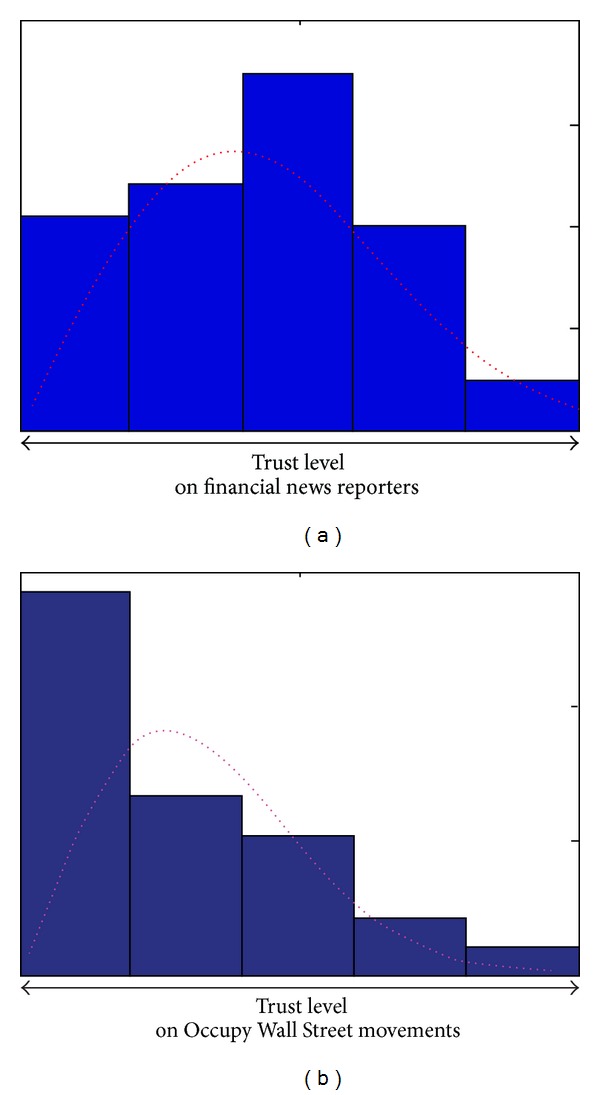
John Hancock survey of American investors' thoughts concerning trust.

Trust is usually considered as a belief or a cognitive stance that is finally quantified by a subjective probability [5]. In many industries, the trust level is usually qualified by multiple evaluations among transaction partners [27]. For example, the interval trust investigation method has been adopted by John Hancock Company, a financial investigation company operating in the USA and Canada, for more than ten years. Their questionnaires assess the investors' trust on financial news reporters and occupy Wall Street movements. Each respondent's evaluation of trust is collected and depicted by an implicit subjective probability (Figure 1). Motivated by the industrial practice, we consider trust as a random variable, whose distribution function is known.

In many cases, trust level is decided by the trustee's reputation [28]. Note that reputation is gradually built up, based on past experiences, on a set of criteria [2]; a good reputation usually means a high level of trust [16]. Actually, trust is also determined by intuitions, which can be specified as social norms, environment, the decision makers' personalities, and so forth [17]. In this paper, the supplier gets demand information from two channels: his own forecast and the retailer's report. When the demand information from these two channels do not match (demand gap), the supplier does not fully trust the demand information from any single channel from his intuition. Therefore, the supplier's trust results from two facts: the retailer's reputation and demand gap.

Based on the above analysis, we take trust value *T* as a random variable which is shaped by retailer's reputation and demand gap. The p.d.f. (probability density function) of trust value *T* can be denoted by *T* ~ *f*(*t*∣*R*, Δ), where 0 ≤ *t* ≤ 1, *R* is the retailer's reputation, and Δ is demand gap.

In this context, market demand is formulated by *D* = *μ* + *ε*, where *μ* is a positive constant denoting the average market demand and *ε* describes the demand fluctuation. Both the retailer and the supplier forecast the value of *μ*, respectively, while they both know that *ε* is a random variable and its cumulative function is Γ(*ε*) and probability density function is *τ*(*ε*). Although the retailer forecasted demand is *μ*_*R*_, she reports the demand information *μ*_*RS*_ to the supplier. A fully trusting supplier implicitly believes the reported demand. However, a supplier who is not fully trusting uses the reported demand *μ*_*RS*_ to update his belief about the market demand. Let *μ*_*S*_ be the supplier forecasted demand; the demand gap is Δ = |*μ*_*RS*_ − *μ*_*S*_|/*μ*_*S*_. Obviously, trust level *T* increases with reputation *R* and decreases with demand gap Δ. The supplier fully believing in and the supplier not fully believing in retailer reported information are two special cases, where the corresponding trust values are 1 and 0, respectively, [1]. Thus, the range of *T* is [0,1]. Since *T* is a random variable, Pr(*T* ≤ *b*∣*R*, Δ) is the possibility that the supplier's trust in the trustee is lower than *b*.

As reputation is important in business, a retailer with an excellent reputation can gain a high level of trust from the supplier. Because the trust level *T* is a random variable and increases with the retailer's reputation, we have

(1) Pr(T<b ∣ R1,Δ)<Pr(T<b ∣ R2,Δ), where  R1>R2.

Similarly, a supplier would like to trust the retailer whose reported demand information is identical with this forecasting. Thus, we have

(2) Pr(T<b ∣ R,ΔS1)<Pr(T<b ∣ R,ΔS2),where  ΔS1<ΔS2.

According to (1), a low-reputation retailer, agreeing with the supplier on the average demand, can also be highly trusted. The trust evaluation model based on reputation and demand gap is represented by Example 1.

**Example 1 .**

A beta probability function can be used to represent probability distributions of binary events, and posteriori probabilities of binary events can be represented as beta distributions [29]. The expression for posteriori probability estimates of binary events can be found in probability theory books (e.g., [30]). In this paper, beta distribution *B*(*α*, *β* + *γ*Δ_*S*_) is used to present the supplier's trust, which is indexed by parameters *α* and *β* + *γ*Δ_*S*_. *α* and *β* are shape parameters and Δ_*S*_  (Δ_*S*_ = |*μ*_*RS*_ − *μ*_*S*_|/*μ*_*S*_) is the demand gap between the reported demand *μ*_*RS*_ and the supplier's forecasted demand *μ*_*S*_. *γ* can be taken as the contribution margin of the demand gap to supplier's evaluation of trust. The two scenarios where the supplier fully trusts and fully distrusts the retailer reported demand are special cases of trust distribution. Thus, if the supplier fully trusts the retailer reported demand, the trust value is *B*(*α*, *β* + *γ*Δ_*S*_∣*α* → +*∞*) → 1, but if the supplier fully distrusts the retailer reported demand, the trust value is described as *B*(*α*, *β* + *γ*Δ_*S*_∣*β* + *γ*Δ_*S*_ → +*∞*) → 0. When the demand gap equals zero, the p.d.f. of the supplier's trust is *f*(*t*_0_∣*R*, 0) = *B*(*α*, *β*). In this situation, *f*(*t*_0_∣*R*, 0) = *B*(*α*, *β*) stands for the retailer's reputation induced trust, which can be called initial trust. In numerical experiments, it is supposed that the demand fluctuation value *ε* follows a normal distribution: *N*(0,16). The given variables are *p*_*S*_ = 20, *p*_*R*_ = 40, *c* = 10, *b* = 0.7, *α* = 4, *β* = 0.5, *γ* = 0.5, and *c*_*k*_ = 3.

**Observation 1 (maintaining a certain trust level is costly for a few negative experiences usually lead to a big loss of trust).**

**Figure 2:**
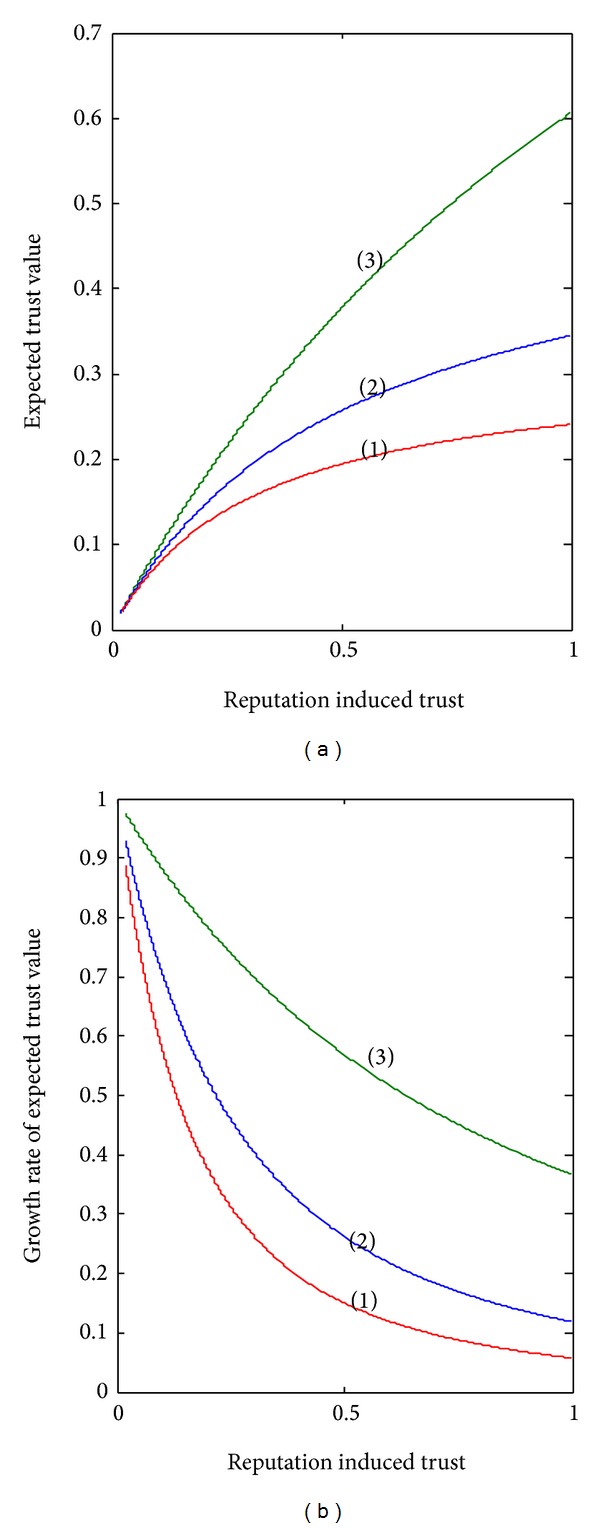
Reputation and the expected trust value.

In order to find out how a retailer's reputation affects supplier's trust, we first fix the value of demand gap and then observe how the supplier's trust changes along with the initial trust (reputation leaded trust). Three group experiments are carried out where the demand gaps are set at 0.13, 0.38, and 0.63.  Figure 2(a) shows that the mean of supplier's trust *E*[*B*(*α*, *β* + Δ*r*)] increases with the retailer's initial trust reputation: *E*[*B*(*α*, *β*)]. It means a retailer of good repute can easily be trusted by the supplier. This is obviously reasonable and common in the industrial world. In Figure 2(a), given initial trust, the smaller demand gap means a higher trust level. Considering a supply chain with more than one retailer and with the retailers having similar reputations, the supplier leans on the retailer whose reported demand is closer to the supplier's forecasting. This is why common sense is very important in cooperation between companies.

The growth rates of the curves in Figure 2(a) are drawn in Figure 2(b) and show that the growth rates decrease with the retailer's initial trust. Supposing three retailers have the same reputation, the initial trust is equal according to Figure 2(b). However, for the retailer whose demand gap is smaller, the growth rate is higher. This phenomenon can be explained as follows: “it takes a lot of trust-positive experiences to gain trust; it takes only a few trust-negative experiences to lose trust” [31].

**Figure 3:**
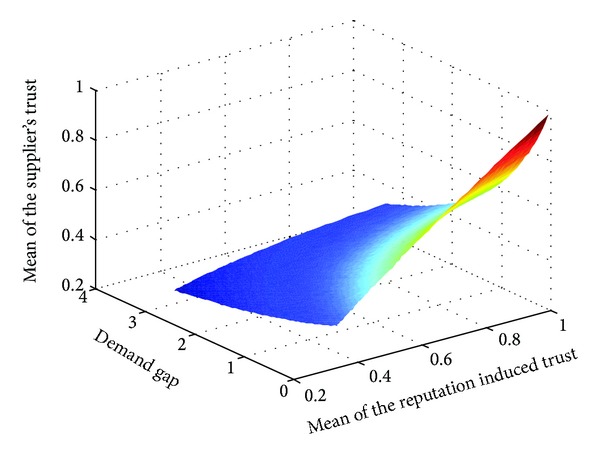
The relationship between reputation induced trust, demand gap, and supplier's trust.

We represent the demand gap, the mean of initial trust, and the mean of supplier's trust on retailer reported demand (supplier's trust for short) in Figure 3. It shows that the supplier's trust increases with initial trust (reputation induced trust) and decreases with the demand gap. When a retailer's reputation is high and the demand gap is low, the supplier easily trusts her reported demand information. At the same time, if a retailer with low reputation (the new entrants or retailers with bad performances in the past) reports an identical demand with the supplier's forecast, she can also achieve a high level of trust.

### 3.1. Trust Level from the Supplier's Perspective

In the industrial world, the retailer is close to the market and knows more about the market demand, so a retailer's forecasted demand *μ*_*R*_ is usually more precise than the supplier's forecasted demand *μ*_*S*_. However, in order to maximize her profit, the retailer reports the demand as *μ*_*RS*_ rather than her forecasted average demand *μ*_*R*_ to the supplier. In other words, the variables *μ*_*R*_, *μ*_*RS*_, and *μ*_*S*_ may not be equal to each other. In the interaction, the retailer intends to distort forecasted demand (see *(A.3)*), which sometimes induces the supplier to build a larger capacity. However, the supplier's trust level decreases with Δ_*S*_, and the distorted demand information leads to a low trust level which, in turn, leads the supplier to set up a low capacity considering the demand uncertainty. Thus, the retailer makes a decision on the reported demand considering these two situations. Since the supplier's trust decreases with the forecasting gap Δ_*S*_, the supplier's trust can be written as

(3) TS~f(t ∣ R,ΔS), where  ΔS=(μRS−μS)μS.

Trust is supplier's willingness to rely on the retailer's forecast report to determine capacity [1]. The supplier relies on the forecasted demand and the retailer reported demand to finally evaluate market demand. If he fully trusts the reported demand, the trust value on the reported demand becomes a constant value 1, and his trust on his forecasted demand is zero. Conversely, if he completely distrusts the reported demand, his trust on his own forecasted demand is 1. In this paper, the supplier's trust value on the retailer's reported demand *μ*_*RS*_ is denoted by *T*_*S*_; thus, the trust value on his own forecasted demand *μ*_*S*_ is (1 − *T*_*S*_) [14]. As suggested by Clemen and Winkler [32], we assume that the supplier combines the two demands of *μ*_*RS*_ and *μ*_*S*_ using a simple weighted average. Therefore, the supplier's evaluation of the market demand can be written as

(4) DS=TSμRS+(1−TS)μS+ε.

In (4), *T*_*S*_ *μ*_*RS*_ + (1 − *T*_*S*_)*μ*_*S*_ is the supplier's evaluation of average market demand, which is also a positive random variable. *ε* is a zero-mean random variable with p.d.f. as *τ*(*ε*). Thus, the supplier's evaluation of market demand is *D*_*S*_.

### 3.2. Trustworthiness Level from the Retailer's Perspective

The notations *μ*_*S*_ and *μ*_*R*_ represent the supplier and the retailer's private information, respectively. Though the retailer does not know the specific value of *μ*_*S*_, she can employ the concept of Bayes' rule to evaluate the value. The retailer forecast of the average market demand is *μ*_*R*_, so she believes that the supplier's private forecast information ${\hat{\mu }}_{S}$ is arrived at by Bayes' rule. In other words, the p.d.f. of ${\hat{\mu }}_{S}$ can be written as $\phi ({\hat{\mu }}_{S}|\mu_{R})$ (where ${\hat{\mu }}_{S}\geq 0$). Hence, from the retailer's prospective, the demand gap can be written as $\Delta_{R}=\left|\mu_{RS}-{\hat{\mu }}_{S}\right|/{\hat{\mu }}_{S}$. Therefore, the retailer's evaluation of the trustworthiness level is

(5) TR~∫0+∞f(t ∣ R,ΔR)ϕ(μ^S ∣ μR)dμ^S,     where  ΔR=|μRS−μ^S|μ^S.

From (5) we can find that *T*_*R*_ is a random variable and its distribution function is shaped by retailer forecasted demand *μ*_*R*_, the reported average demand *μ*_*RS*_, and her evaluation of the supplier's forecasted demand ${\hat{\mu }}_{S}$.

According to (4) and (8), trust, from the supplier's perspective, may not be the same from the retailer's perspective.  Example 2 is thus employed to examine the difference.

**Example 2 .**

We suppose that the demand fluctuation value *ε* follows a normal distribution *n*(0,16). The given variables are *p*_*S*_ = 20, *p*_*R*_ = 40, *c* = 10, *b* = 0.7, *α* = 4, *β* = 0.5, *γ* = 0.5, and *c*_*k*_ = 3. The retailer estimates the value of ${\hat{\mu }}_{S}$ utilizing Bayes' rule ${\hat{\mu }}_{S}\sim \phi ({\hat{\mu }}_{S}|\mu_{R})$. We assume ${\hat{\mu }}_{S}$ belongs to a normal distribution ${\hat{\mu }}_{S}\sim n(\mu_{R},\Delta_{\mu_{s}}^{2})$, where its mean is ${\hat{\mu }}_{S}$ and its variance is Δ_*μ*_*s*__^2^. The confident retailer believes *μ*_*S*_ has fallen into a small range, which means Δ_*μ*_*s*__^2^ is a small value at this situation.

**Observation 2 (a confident retailer always evaluates a high trustworthiness level).**

**Figure 4:**
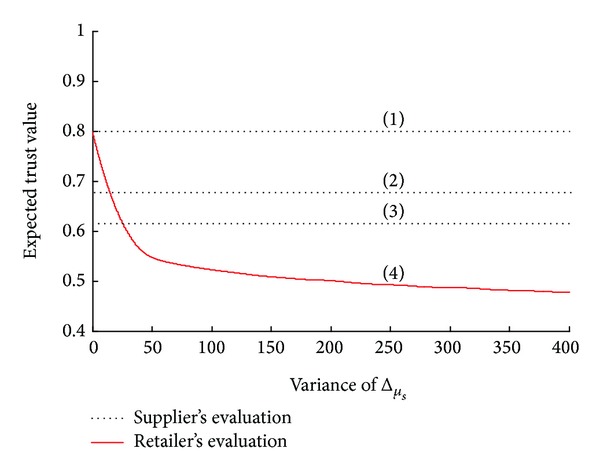
Mean of trust from the supplier's and retailer's perspectives.

Supposing three situations of the supplier's forecasted average demand *μ*_*S*_ are 70, 65, and 60 and the retailer reported average demand *μ*_*RS*_ is 70, the means of the supplier's trust are depicted by the straight lines (1), (2), and (3). It can be found that when the variance Δ_*μ*_*s*__^2^ = 0, the supplier's trust is 0.8, 0.68, and 0.62, respectively. However, a retailer lacking confidence evaluates *μ*_*S*_ in a large boundary; thus, the corresponding variance Δ_*μ*_*s*__^2^ is big. Therefore, when the retailer reported demand is *μ*_*RS*_ = 70, the red curve in Figure 4 shows the retailer's evaluation of mean trust level along with the Δ_*μ*_*s*__^2^. It is found that the retailer's evaluation of the level of trustworthiness *E*(*T*_*R*_) decreases with Δ_*μ*_*s*__^2^. When the Δ_*μ*_*s*__^2^ = 20, we can deduce that *E*(*T*_*R*_∣*μ*_*RS*_ = 70, Δ_*μ*_*s*__^2^ = 0) > *E*(*T*_*R*_∣*μ*_*RS*_ = 70, Δ_*μ*_*s*__^2^ = 20) = *E*(*T*_*S*_∣*μ*_*S*_ = 65). This indicates two aspects: (1) the confident retailer may overestimate her level of trustworthiness; (2) a retailer lacking in confidence may have an identical evaluation regarding the trustworthiness level of the supplier.

## 4. Distrust-Induced Perceived Risk Management

Trust is inherently linked to perceived risk [7]. A supplier who fully trusts the retailer reported demand information will not anticipate any risks. Once the supplier does not fully trust the reported demand information, he perceives negative outcomes (perceived risk) resulting from the uncertainty of the demand information [33]. Risk premium tools are put in place to counteract the risks that the decision makers face. For example, an insurance company charges a higher premium rate from a high-risk client [34], and similar risk hedging methods are widespread in the financial industry, investment industry, and so forth. The risk mitigation approaches include insurance, options, deposit, risk-pooling on costs, or profit-sharing contracts [21]. The main intention of these risk management approaches is the same, that is, “risk management through coordination or collaboration among the supply chain partners in order to ensure profitability and continuity” [35]. Because perceived risk results from the supplier's distrust and the deposit contract is a familiar tool for pooling risks in an uncertain environment, we propose a trust-based deposit contract for the supplier to decrease his perceived risks. In this paper, the more the supplier distrusts the retailer's information, the more deposit is supposed to be charged. The two extreme cases are where the supplier fully trusts and fully distrusts the retailer's reported demand information. When the supplier fully trusts the retailer's information, the supplier will not need the retailer to share the prepared capacity costs. Contrarily, once the supplier fully distrusts the retailer's information, he charges a high deposit from the retailer (denoted by *m*). The risk management is carried out in a two-stage negotiation.

Although, the deposit, as the risk mitigation approach, is adopted in this paper, other approaches (such as options, insurances) can also be adopted to mitigate the perceived risk. If these approaches are used, the supplier and the retailer negotiate the option, insurance, risk-pooling, or profit-sharing contract at the first stage and then make decisions at the next stage, based on the contract in the former stage. In this paper, we focus on introducing a new concept of the trust based on a psychological perspective and on describing a two-stage negotiation approach to mitigate distrust-induced perceived risks.

### 4.1. Deposit Contract

Forecast sharing can be induced through contracts [5]. These approaches are shown to enable credible forecast information sharing. Here in this paper, we are primarily interested in understanding how the supplier mitigates the distrusted induced perceived risk and how the risk mitigation approach proceeds. Even a simply risk management tool enables us to focus on the forecast sharing problem with one-time interaction. Therefore, we use an unpayback deposit as the perceived risk management tool.

**Figure 5:**
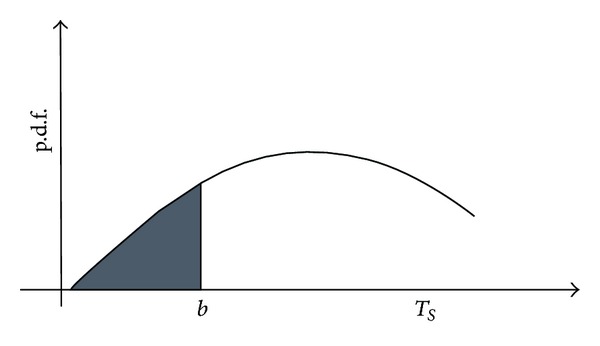
The p.d.f. of supplier's trust.

In an uncertain circumstance, the decision maker adopts different solutions for different risk levels according to his/her trust threshold [36]. It is common and logical that the truster will only engage in a transaction on condition that the level of trust exceeds some threshold [37]. The threshold is dependent on the transaction context and on both parties' negotiations of the transaction. For simplicity, the threshold (denoted by *b*) is a given constant in this paper and we do not show more details about how this constant is calculated. Thus, Pr(*T* ≤ *b*∣*R*, Δ_*S*_) is the possibility of the trust value being in the range [0, *b*] (Figure 5), which reflects the supplier's degree of distrust. Obviously, Pr(*T* ≤ *b*∣*R*, Δ_*S*_) decreases in tandem with the supplier's trust level. Since the deposit is *m* per product when the supplier fully distrusts the retailer reported information, the deposit per product for a certain trust level *T* ~ *f*(*t*∣*R*, Δ_*S*_) is specified as *m*Pr(*T* ≤ *b*∣*R*, Δ_*S*_) (deposit rate).

The threshold *b* is public information for the supplier and the retailer; since they have different evaluations about the trust level (4) and (8), the supplier and the retailer have different evaluations of the deposit rate at stage 1. The larger retailer reported demand means the larger perceived risk for the supplier. So the larger deposit is charged. The deposits from the supplier and retailer's view are

(6) μRSmPr(T≤b ∣ R,ΔS)=μRSm∫0bf(t ∣ R,ΔS)dt,          where  ΔS=|μRS−μS|μS,μRSmPr(T≤b ∣ R,ΔR)  =μRSm∫0b∫0+∞f(t ∣ R,ΔR)ϕ(μ^S)dμ^Sdt,         where  ΔR=|μRS−μ^S|μ^S.

**Theorem 3 .**

If the reported demand is equal to the supplier forecasted demand, the retailer's evaluation of the deposit rate is greater than that of the supplier's evaluation.

**Proof**

According to the assumption in Theorem 3, if *μ*_*S*_ = *μ*_*RS*_, we get Δ_*S*_ = |*μ*_*RS*_ − *μ*_*S*_|/*μ*_*S*_ = 0. The deposit rate from the supplier's perspective can be written as *m*Pr(*T* ≤ *b*∣*R*, Δ_*S*_) = *m*Pr(*T* ≤ *b*∣*R*, 0).

Similarly, the retailer calculates the deposit rate based on her evaluation of the supplier's forecasted average demand ${\hat{\mu }}_{S}$. Her evaluation of the deposit rate is *m*Pr(*T* ≤ *b*∣*R*, Δ_*R*_). In terms of (2), we have

(7) mPr(T≤b ∣ R,ΔR)≥mPr(T≤b ∣ R,0)=mPr(T≤b ∣ R,ΔS).

Theorem 3 shows that the retailer overevaluates the deposit rate (the loss resulting from distrust), even when her reported demand equals the supplier's forecasted demand. The retailer does not know the specific value of *μ*_*S*_; this is why the retailer sometimes overevaluates the deposit rate.

**Example 4 .**

In order to compare the deposit from the retailer and supplier's perspectives, we use the variables and their values that were used in Example 2 to carry out the following experiment.

**Observation 3 (the deposit rates from the retailer's and supplier's perspectives differ).**

**Figure 6:**
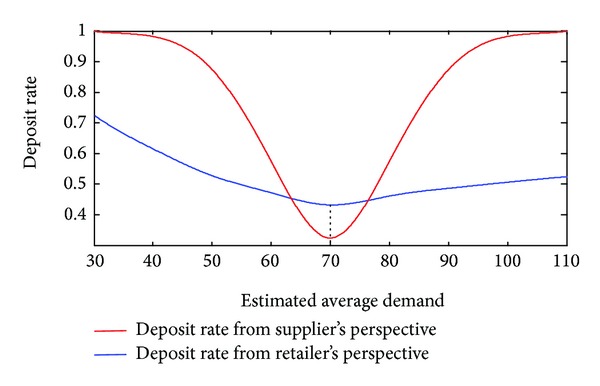
The deposit rates from supplier's and retailer's perspectives.

The reported demand is set at *μ*_*RS*_ = 70. The red curve in Figure 6 shows that the deposit rate changes along with the supplier's forecasted demand *μ*_*S*_. When *μ*_*S*_ = *μ*_*RS*_ = 70, which means that the demand gap equals zero, the supplier's deposit rate is at its lowest rate. It is reasonable to expect that the supplier trusts the retailer. Given a fixed value of Δ_*μ*_*s*__ = 30, the deposit rate, changing with the mean of $E({\hat{\mu }}_{S})$ from the retailer's perspective, is indicated by a blue curve. The blue curve shows that the deposit rate changes with her forecasted demand *μ*_*R*_ when the retailer's reported demand is fixed at *μ*_*RS*_ = 70. When the retailer exactly reports her forecasted demand *μ*_*R*_ = *μ*_*RS*_, the deposit rate from the retailer's perspective is at its lowest level. The supplier and retailer differently evaluate the deposit rate at stage 1; they make decisions at this asymmetrical point.

### 4.2. Trust-Based Perceived Risk Management with Deposit Contract

The supplier's capacity decision is a response to the retailer's reported demand [38]. In order to maximize her profit, the retailer is motivated to overstate the market demand, primarily through contracting between the supply chain partners by, for example, quantity discount contract, buy-back contract, and so on. In this paper, we use the deposit contract to align the supply chain partner's objectives. The less the supplier trusts the retailer's reported demand, the more deposit is expected by the supplier. However, the retailer can refuse the deposit contract once the profit in the deposit contract is less than that without the deposit contract.

The deposit by the retailer can help the supplier to pool the potential loss when he does not fully trust the retailer reported demand. At the same time, the retailer can benefit from the decentralized decision making pattern (see the appendix) without the contract. In order to maximize profit, the supplier and retailer negotiate the deposit rule at the first stage. After the deposit rule is made, the supplier and the retailer make the reported demand and the capacity accordingly. Actually, this two-stage negotiation process is like the interaction between an insurance company and its customers. The insurance company first proposes some optional insurance packages or items to a customer. Then the customer chooses the most suitable package or items; sometimes there is negotiation. Once the insurance company and the customer initially agree on the insurance contract, then the next step for the customer is to pay the premium and carry out the contract.

**Figure 7:**
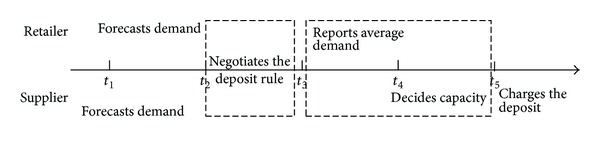
Sequence of events.

In a supply chain with one supplier and one retailer, events that happen over time are shown as in Figure 7. The prepared capacity and the reported demand quantity are both decision variables and are determined by the supplier and the retailer, respectively. The retailer first reports the demand to the supplier, and then the supplier makes the decision about capacity based on the reported demand and his own forecasted demand.

In this paper, the decisions can be formulated as two-stage negotiations based on the sequence of events. At stage one, the supplier and the retailer negotiate the deposit rate for one unit of prepared capacity. The more a supplier distrusts the reported demand, the more he feels the risk. Thus, he pools the perceived risks with a higher deposit rate for one unit of prepared capacity. At stage two, the retailer and the supplier make decisions about reported demand quantity and the prepared capacity by means of a Stackelberg game. To balance the two stages, we proceed with backward induction, where we first solve the problems in stage two and then optimize the decisions in stage one. Note that once the deposit rule is fixed via negotiation in stage one, the deposit towards one unit capacity will be charged at stage two.

*Stage 2 (the reported demand and the optimal product quantity with given deposit rate).* At the first stage, the supplier and the retailer negotiate the deposit index *m*. Based on the known value of deposit index *m*, the retailer and supplier decide on the reported demand and production quantity sequentially at stage two. The decision processes can be considered as a Stackelberg game. Therefore, we solve the supplier's profit model first.

(*1) Supplier's Profit Model.* After receiving the retailer reported demand *μ*_*RS*_, the supplier calculates his evaluated demand according to (3). Thus, the supplier's profit function is modeled as

(8) ΠS(Q)=Eε,DS[(ps−c)min⁡(Q,DS)−ckQ]+μRSmPr(T≤b ∣ R,ΔS),where  μRSmPr(T≤b ∣ R,ΔS)=μRSm∫0bf(t ∣ R,ΔS)dtΔS=(μRS−μS)μS.

Extant literature on forecast sharing and supply chain coordination implicitly assumes that supply chain members either absolutely trust each other or do not trust each other at all. Contrary to this all-or-nothing perspectives, a continuum existing between these two extremes is proposed by the trust model (4) and employed in supplier's profit function (8). Because *T*_*S*_ and *ε* are random variables, *D*_*s*_ is also a random variable and its p.d.f. and c.d.f. (cumulative distribution function) are denoted by *g*_*S*_(·) and *G*_*S*_(·). Thus, *g*_*S*_(*D*_*S*_) ≥ 0 and 0 ≤ *G*_*S*_(*D*_*S*_) ≤ 1. The supplier's profit function can be transformed as follows:

(9) ΠS(Q)=(ps−c)[∫0QxgS(x)dx+∫Q+∞QgS(x)dx]−ckQ+μRSm∫0bf(t ∣ R,ΔS)dt.

The first part of the right side of (9) is a polynomial of *Q*, so derivative and second-derivative of the polynomial on *Q* are 1 − *G*_*S*_(*Q*) and −*g*_*s*_(*Q*), respectively. Because 1 − *G*_*S*_(*Q*) ≥ 0 and −*g*_*S*_(*Q*) ≤ 0, the polynomial (*p*_*s*_ − *c*)[∫_0_^*Q*^ *xg*_*S*_(*x*)*dx* + ∫_*Q*_^+*∞*^ *Qg*_*S*_(*x*)*dx*] is concave in *Q*. The second part of the right hand of (9), −*c*_*k*_ *Q* + *μ*_*RS*_ *m*∫_0_^*b*^ *f*(*t*∣*R*, Δ_*S*_)*dt*, is a linear polynomial of *Q*; thus, it is also concave in *Q*. Because the sum of concave functions is also a concave function, (16) is a concave function of *Q*. That means the optimal solution of *Q* exists.

The derivative of Π_*S*_(*Q*) by *Q* can be calculated as

(10) dΠS(Q)dQ =−ck+(pS−c)  ×[1−∫01Γ(Q+tμRS−μRS−tμS)f(t ∣ R,ΔS)dt].

Because Δ_*S*_ = |*μ*_*RS*_ − *μ*_*S*_|/*μ*_*S*_, the polynomial ∫_0_^1^Γ(*Q* + *tμ*_*RS*_ − *μ*_*RS*_ − *tμ*_*S*_)*f*(*t*∣*R*, Δ_*S*_)*dt* is denoted by *U*(*Q*∣*μ*_*RS*_, *μ*_*S*_). If the value of (10) equals zero, the supplier's optimal decision of *Q* is

(11) Q∗=U−1(pS−c−ckpS−c ∣ μRS,μS).

Note that Q* is a function of *μ*_*S*_ which is the supplier's private information, so the retailer knows that the supplier decides his capacity by (11). Because the retailer evaluates *μ*_*S*_ with ${\hat{\mu }}_{S}$, the retailer's evaluation about the capacity is

(12) QR∗=U−1(pS−c−ckpS−c ∣ μRS,μ^S).

(*2) Retailer's Profit Model.* Because of $T_{R}\sim \int_{0}^{+\infty }f(t|R,\Delta_{R})\phi ({\hat{\mu }}_{S}|\mu_{R})d{\hat{\mu }}_{S}$ (3), the retailer's expected profit can be formulated by

(13) ΠR(μRS)=Eε[(pR−pS)min⁡⁡(QR∗,μR+ε)]−μRSmPr(T≤b ∣ R,ΔR),where  μRSmPr(T≤b|R,ΔR)  =μRSm∫0b∫0+∞f(t ∣ R,ΔR)ϕ(μ^S)dμ^SdtΔR=|μRS−μ^S|μ^S.

In (13), the retailer's expected profit consists of two parts: the selling revenue and the deposit. In the supplier dominated supply chain, the supplier and the retailer negotiate the deposit index *m* firstly, which is correlated to the retailer's reported demand *μ*_*RS*_. Thus, optimal *m*(*μ*_*RS*_) at stage 1 is a function of the retailer reported demand *μ*_*RS*_, written as *m*(*μ*_*RS*_). Note that the retailer can earn *p*_*R*_ − *p*_*S*_ by selling one unit of product; the deposit she is willing to be charged by supplier is less than *p*_*R*_ − *p*_*S*_; that is, *m*Pr(*T* ≤ *b*∣*R*, Δ_*R*_) ≤ *p*_*R*_ − *p*_*S*_. We introduce the deposit index *m*(*μ*_*RS*_) into (13) and get the optimal reported demand *μ*_RS_*:

(14) μRS∗=argμRSmax⁡ΠR(μRS ∣ m(μRS)).

*Stage 1* (the negotiation of the deposit rule). At stage two, the deposit index *m*(*μ*_*RS*_) is already known. The supplier and retailer then make their decisions on Q* and *μ*_RS_*, respectively, by means of a Stackelberg game. At stage one, the supplier and the retailer negotiate about the deposit index *m*(*μ*_*RS*_) in the information asymmetry circumstance.

According to (3), when the retailer reported demand is *μ*_RS_*, the supplier's trust level is *T*_S_*(*μ*_RS_*) ~ *f*(*t*∣*R*, Δ_S_*). Therefore, the deposit that the supplier claims from the retailer is *μ*_RS_*mPr(*T* ≤ *b*∣*R*, Δ_S_*) and the supplier evaluates the market demand to be *D*_S_* = *T*_S_*μ_RS_* + (1 − *T*_S_*)*μ*_*S*_ + *ε*. Thus, the supplier's expected profit can be formulated by

(15) ΠS∗(m) =ETS∗,ε{(pS−c)min⁡[Q∗,TS∗μRS∗+(1−TS∗)μS+ε]  −ckQ∗+μRS∗mPr(T≤b ∣ R,ΔS∗)}.

In (15), variable *ε* and variable *T*_S_* are random. The supplier's expected profit under the risk management contract should be more than that in the decentralized supply chain pattern. Otherwise, the supplier makes decision under the decentralized pattern. Because the supplier's expected profit under the decentralized pattern is ${\bar{\Pi }}_{S}^{*}$ (see *(A.7)* in the appendix), supplier's threshold of joining in the risk management contract is $\Pi_{S}^{*}(m)\geq {\bar{\Pi }}_{S}^{*}$.

Similarly, from the perspective of the retailer, the trust value is *T*_R_* ~ *f*(*t*∣*R*, Δ_R_*) and the deposit value is *μ*_RS_*mPr(*T* ≤ *b*∣*R*, Δ_R_*). Thus, the retailer's expected profit is

(16) ΠR∗(m)=Eε,ΔR∗[(pR−pS)min⁡(QR∗,μR+ε)−μRS∗mPr(T≤b ∣ R,ΔR∗)],

where *ε* ~ *τ*(*ε*), $\Delta_{R}^{*}=\left|\mu_{RS}^{*}-{\hat{\mu }}_{S}\right|/{\hat{\mu }}_{S}$, and ${\hat{\mu }}_{S}\sim \phi ({\hat{\mu }}_{S}|\mu_{R})$.

Equation (16) is the expected profit from the retailer's perspective if she accepts the deposit contract at stage 1. However, if the retailer does not accept the deposit contract, the retailer's expected profit ${\bar{\Pi }}_{R}^{*}(m)$ can be calculated by *(A.8)* in the appendix. In the supplier leaded supply chain, the retailer's bottom line in the negotiation is that the profit in the deposit contract is not less than that in the decentralized pattern. Thus, $\Pi_{R}^{*}(m)\geq {\bar{\Pi }}_{R}^{*}$.

Note that the constraints $\Pi_{S}^{*}(m)\geq {\bar{\Pi }}_{S}^{*}$ and $\Pi_{R}^{*}(m)\geq {\bar{\Pi }}_{R}^{*}$ are the bottom lines for the supplier and the retailer to join in the deposit contract; we named them as join-in constraints. In stage one, the supplier's objective is maximizing his profit under his join-in constraint:

(17) m∗(μRS∗)=argmax⁡mΠS∗(m)S.t. ΠR∗(m)≥Π¯R∗  ΠS∗(m)≥Π¯S∗.

The negotiated deposit index m*(*μ*_RS_*) is calculated according to (17). However, it is possible that the join-in constraints cannot be simultaneously satisfied. So, if there is no solution of (17), the supplier and the retailer make their decisions in the decentralized pattern.

## 5. Numerical Study

According to (3), the reported demand *μ*_*RS*_ determines the distribution of supplier and retailer evaluated trust *t*_*S*_ and *t*_*R*_. However, the supplier's evaluation of market demand and his decision on deposit are made based on trust distribution, so the objective model is complex, making it hard to find the optimal solution by mathematical derivation. In this study, some observations are found by numerical simulations. In numerical experiments, the demand fluctuation value *ε* follows a normal distribution: *n*(0,16). The given variables are *p*_*S*_ = 20, *p*_*R*_ = 25, *c* = 10, *b* = 0.2, *α* = 2, *β* = 1, *γ* = 3, *μ*_*R*_ = 105, *c*_*k*_ = 3, and *μ*_*S*_ = 90. Although the retailer does not know the supplier's forecast of average market demand *μ*_*S*_, she evaluates it based on her forecasted demand *μ*_*R*_ = 105 as a uniform distribution ${\hat{\mu }}_{S}\sim U(\mu_{R}-20,\mu_{R}+20)=U(85,125)$.

At stage one, the supplier and the retailer negotiate about the deposit index *m*. Once *m* is determined by negotiation at stage one, the retailer makes the decision on reported demand *μ*_*RS*_ and then the supplier decides the optimal capacity at stage 2. The backwards decision processes are presented as follows.

(*1) Decisions at Stage 2.* The deposit index *m* is already determined at stage one, so the values of the parameters are introduced into (9); the supplier's profit function can be written as follows:

the supplier's profit function:

(18) ΠS(Q)=Eε,DS[10min⁡(Q,DS)−3Q]+μRSmPr(T≤0.2 ∣ R,ΔS),where  μRSmPr(T≤0.2 ∣ R,ΔS)  =μRSm∫00.2f(t ∣ R,ΔS)dtΔS=|μRS−90|90.

Note that the *μ*_*S*_ is the supplier's private information, which means the retailer evaluates the Δ_*S*_ to be $\left(\mu_{RS}-{\hat{\mu }}_{S}\right)/{\hat{\mu }}_{S}$. So the retailer evaluates the supplier's profit as $\Pi_{SR}(Q|{\hat{\mu }}_{S})$ to be

(19) ΠSR(Q)=Eε,D^S[10min⁡(Q,D^S)−3Q]+μRSmPr(T≤0.2 ∣ R,ΔR),where  μRSmPr(T≤0.2 ∣ R,ΔR)  =μRSm∫00.2∫85125ϕ(μ^S)140dμ^Sf(t ∣ R,ΔR)dtD^S=TRμRS+(1−TR)μ^S+ε,ΔR=(μRS−μ^S)μ^S.

Although the supplier's evaluated the demand to be *D*_*S*_, the retailer does not know the value of *μ*_*S*_ and her evaluation about *D*_*S*_ is ${\hat{D}}_{S}$ in (19). Meanwhile, the retailer's profit function is

(20) ΠR(μRS)=Eε[5min⁡(QR∗,105+ε)]−μRSmPr(T≤0.2 ∣ R,ΔR)where  μRSmPr(T≤0.2 ∣ R,ΔR)  =μRSm∫00.2∫85125ϕ(μ^S)140f(t ∣ R,ΔR)dμ^SdtΔR=|μRS−μ^S|μ^S.

In the Stackelberg game at stage one, the retailer makes the decision of *μ*_*RS*_ firstly, and then the supplier makes the decision on its capacity *Q*. Thus, once the retailer's optimal decision on *μ*_*RS*_ is made, the supplier's capacity decision is

(21) Q∗=47.0586+0.49635μRS+0.006544mμRS.

Equation (21) indicates that the supplier's optimal capacity increases with deposit index *m* and the retailer reported demand *μ*_*RS*_. Meanwhile, because *μ*_*S*_ is the supplier's private information, the retailer evaluates it as ${\hat{\mu }}_{S}$. Thus, according to (19), the retailer's evaluation of the optimal capacity is

(22) QR∗=11.1309+1.2633μRS−0.1406m2−0.003μRS2.

Note that when the capacity is zero, which means there is no product produced, both the supplier and retailer's profits equal zero. Thus, in this situation they will make decisions under the decentralized pattern and their expected profits are calculated as ${\bar{\Pi }}_{S}^{*}=614.9445$ and ${\bar{\Pi }}_{R}^{*}=500.3709$, respectively, *(A.7)* and *(A.8)*. According to (20), the retailer's profit is the function of *μ*_*R*_, *m*, *μ*_*RS*_, and ${\hat{\mu }}_{S}$. Note that the deposit index *m* is a function of the reported demand *μ*_*RS*_ and is an already known function at stage two, that is, m*(*μ*_*RS*_). Thus, we introduce m*(*μ*_*RS*_) into (20), and then we get the optimal reported demand *μ*_RS_*:

(23) μRS∗=argμRSmax⁡ΠR(μRS).

**Figure 8:**
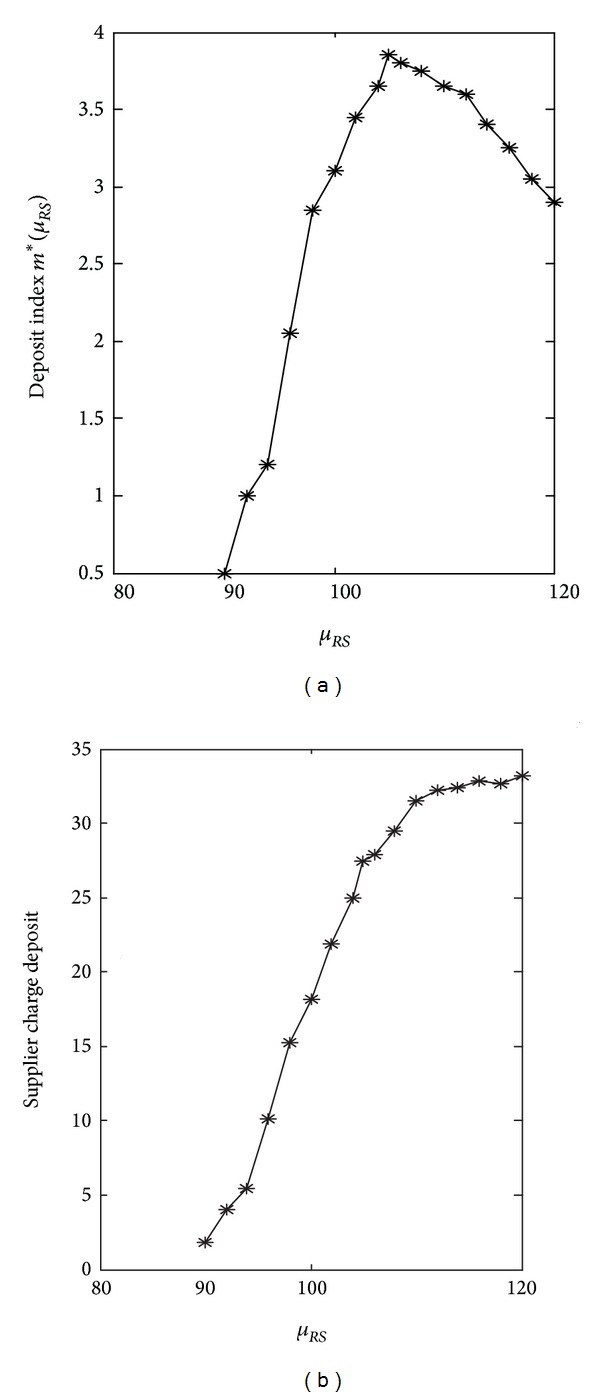
The optimal deposit index m*(*μ*_*RS*_) and the supplier charged deposit.

*Decisions at Stage 1.* Because the retailer's expected profit in the deposit contract is not less than her profit from the decentralized pattern, according to *(A.7)* and *(A.8)*, the supplier and the retailer's bottom lines in the negotiation are Π_*S*_(*m*) > 614.9445 and Π_*R*_(*m*) > 500.3709. Because of (17), $m^{*}=\arg\underset{m}{\max}\prod_{S}(m)$, the optimal deposit index m*(*μ*_*RS*_) and the corresponding deposits are calculated by ten thousand simulations (Figure 8).

The supplier's revenue (9) consists of three parts: the selling revenue, the deposit from the retailer, and the costs. The supplier makes a balance from these three parts when he makes the decision on *m*. Figure 8 shows that optimal deposit index m*(*μ*_*RS*_) is a segmented function of *μ*_*RS*_. m*(*μ*_*RS*_) can be gotten by the responds surface method as

(24) m∗(μRS)={−μRS<90,9.2506+0.001188μRS290≤μRS≤105,7.0126−0.0002869μRS2105≤μRS≤156.34,⟶0μRS>156.34.

Equation (24) shows that the optimal deposit rate m*(*μ*_*RS*_) is a segmented function of *μ*_*RS*_ (see Figure 8). When the retailer reports a demand *μ*_*RS*_, the supplier charged deposit is m*(*μ*_*RS*_)Pr(*T* ≤ 0.2∣*R*, Δ_*S*_)*μ*_*RS*_. Note that the deposit will not be paid back and cannot be more than the retailer's revenue from selling one unit product; that is, m*(*μ*_*RS*_)Pr(*T* ≤ 0.2∣*R*, Δ_*S*_) < *p*_*R*_ − *p*_*S*_ = 5. The segmented function of m*(*μ*_*RS*_) can be analyzed as follows.

(1) When *μ*_*RS*_ < 90: in the decentralized problem, the supplier's profits equals 614.9445. Because the retailer does not know the supplier's private information *μ*_*S*_ and evaluates it as ${\hat{\mu }}_{S}$ and the retailer evaluates her profit in the decentralized pattern as 500.3709, the supplier and retailer's join-in constraints are ${\bar{\Pi }}_{S}^{*}=614.9445$ and ${\bar{\Pi }}_{R}^{*}=500.3709$. When 0 ≤ *μ*_*RS*_ ≤ 90, the supplier and retailer's join-in constraints cannot be simultaneously satisfied, so the supplier and the retailer make their decisions in a decentralized pattern. For example, when *μ*_*RS*_ = 80, the retailer's maximum profit is 446.9478, which is less than the join-in constraint. Thus, there is no solution of the risk management mechanism when *μ*_*RS*_ < 90.

(2) When *μ*_*RS*_ ≥ 90: the supplier takes the retailer reported demand *μ*_*RS*_ into consideration when he does the capacity decisions, and the supplier charged deposit increases with *μ*_*RS*_ (Figure 8). Because *D*_*S*_ decreases with *μ*_*RS*_ (4), the supplier's reliance on the retailer reported demand also decreases. For example, when retailer reported demand is *μ*_*RS*_ = 100, the mean of the supplier's reliance coefficient is mean(*t*_*S*_) = 0.5919. When the retailer reported demand equal to *μ*_*RS*_ = 108, the mean of the coefficient falls down to mean(*t*_*S*_) = 0.5482.

**Figure 9:**
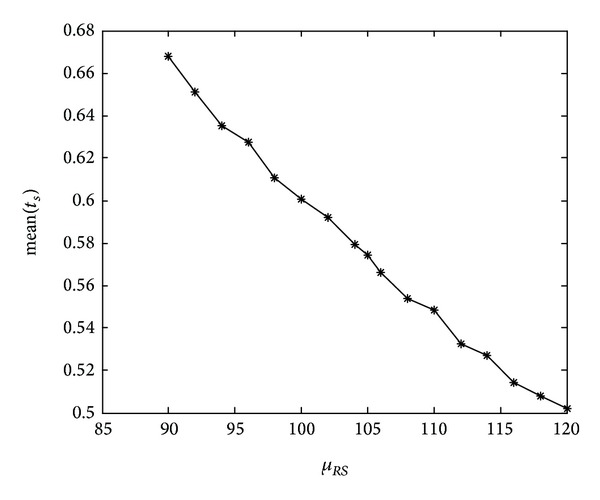
Mean of the supplier's trust changes with *μ*_*RS*_.

Because the supplier's trust level decreases over the retailer's reported demand *μ*_*RS*_ (Figure 9, *μ*_*RS*_ ≥ 90), the supplier's reliance coefficient on the retailer reported demand mean(*t*_*S*_) is getting smaller. However, the supplier's perceived risk increases with *μ*_*RS*_ when *μ*_*RS*_ ≥ *μ*_*S*_. In this situation, the more the demand is reported by the retailer, the more deposit is charged by the supplier (Figure 9).

*Remarks.* The numerical study shows the decision process of the provided perceived risk management. The decision process is preceded in two stages. In Stage 1, both the retailer and supplier negotiate a deposit rule. Based on the deposit rule, the retailer and supplier make decisions by a Stackelberg game in stage 2. According to the existing psychological theories, we assume the supplier's trust follows beta distribution (see Example 1), where shape parameters *α*, *β*, and *γ* are given. Because trust is a type of psychology state, the shape parameters of a firm's trust evaluation model might not be known, which is challengeable in supply chain decision making. Due to this challenge, we can firstly collect the firm's trust level by larger-scale questionnaires. After that, we evaluate the values of the shape parameters based on statistical analysis of the collected data. Based on above analysis, the proposed trust-based supply chain coordination is applicable in industries.

## 6. Conclusions and Potential Researches

In a two-tier supply chain of supplier and retailer, the supplier makes his decision relying on his forecasted demand and the retailer's reported demand. However, it is the retailer's intention to distort information. Thus, the supplier does not fully trust the retailer's reported demand. We first formulate a trust evaluation model based on psychological theory, considering that trust is affected by the retailer's reputation and the demand gap. We find a small demand gap will result in higher trust; it highlights the fact that a firm with low trustworthiness can also earn trust by providing consensus information. Because of information asymmetry, the supplier and retailer value trust differently and we find a confident retailer believing herself is easily trusted. Therefore, overconfident firms are suggested to downgrade its trustworthiness evaluation results. Since trust and perceived risk are inextricably linked together, we suggest a two-stage deposit contract to mitigate the supplier's distrust-induced perceived risk. This risk management tool is applied in the proposed two-stage negotiation process. An example is employed to illustrate the two-stage decision processes and shows that that the proposed two-stage risk mitigate process is practical. Because distrust-induced risk is seldom considered in supply chains, firms can employ the proposed two-stage decision process to align the supply chains.

Without loss of generality, we suggest a general trust quantifying model. Because the trust is a kind of psychology state, different decision makers hold different sharp parameters of trust quantifying models. Thus, the decision makers need to primarily evaluate the sharp parameters before making any decisions in industries. Due to this shortcoming of the proposed model, we will evaluate the sharp parameters by analyzing the industrial data and suggest the corresponding evaluated value of sharp parameters in the extending work. Since decision maker's feeling of trust in supply chain information sharing is seldom considered in supply chain information sharing, investigating trust in supply chain information sharing offers many potential research opportunities. We believe there are at least two major potential research directions in future. One possible research direction is to further analytically study the trust-based decisions in more complex supply chain structures. Since trustworthiness is updated with new experience or new circumstance over time, the other potential research direction is to extend our work in multiperiod transaction problems.

## Figures and Tables

**Table 1:** Notations.

	Supplier		Retailer	
Public information	*p*_*S*_	Supplier's wholesale price	*p*_*R*_	Retailer's retail price *p*_*R*_ < *p*_*S*_
	*c*	Product cost of each product	*T*_0_	Reputation induced being trust 0 < *t*_0_ < 1
	*c*_*k*_	Capacity setup cost of each product	*ε*	Market fluctuation *ε* ~ *τ*(*ε*)

Private information	*μ*_*S*_	Forecasted average demand by supplier →	${\hat{\mu }}_{S}$	Evaluation about $\mu_{S}{\hat{\mu }}_{S}\sim \varphi ({\hat{\mu }}_{S}|\mu_{R})$
			*μ*_*R*_	Forecasted average demand from retailer's perspective

Decision variable	*Q*	Capacity	*μ*_*RS*_	Reported average demand
	*m*	Deposit index		

Evaluated information	Δ_*S*_	The demand gap evaluated by supplier Δ_*S*_ = |*μ*_*RS*_ − *μ*_*S*_|/*μ*_*S*_	Δ_*R*_	The demand gap evaluated by retailer $\Delta_{R}=\left|\mu_{RS}-{\hat{\mu }}_{S}\right|/{\hat{\mu }}_{S}$
	*T*_*S*_	Trust on retailer reported average demand 0 < *t*_*S*_ < 1	*T*_*R*_	Retailer evaluated being trust level 0 < *t*_*R*_ < 1

Objective	${\overset{-}{\Pi }}_{S}$	Supplier's expected profit in the decentralize supply chain	${\overset{-}{\Pi }}_{R}$	Retailer's expected profit in the decentralize supply chain
	Π_*S*_	Supplier's expected profit in the supply chain with the risk management	Π_*R*_	Retailer's expected profit in the supply chain with the risk management

## Appendix

Equilibrium solution in decentralize pattern without risk management contract.

In a decentralized supply chain with one supplier and one retailer, the retailer has the motivation to report unreal demand information to maximize his profit (Theorem A). It is logical that the supplier does not fully trust retailer reported demand information.

Theorem 6 A. Supplier does not fully trust retailer reported demand in decentralized supply chain.

Proof In a decentralized supply chain with one supplier and one retailer, the supplier is assumed to fully trust the supplier reported demand. In this case, the supplier and retailer's expected profit ${\bar{\Pi }}_{S}(Q)$ and ${\bar{\Pi }}_{R}(\mu_{RS})$ can be written as

(A.1) $$\begin{array}{c} {\bar{\Pi }}_{S}\left(Q\right)=\underset{\varepsilon }{E}\left[\left(p_{S}-c\right)\min\left(\mu_{RS}+\varepsilon ,Q\right)-c_{k}Q\right], \end{array}$$

(A.2) $$\begin{array}{c} {\bar{\Pi }}_{R}\left(\mu_{RS}\right)=\underset{\varepsilon }{E}\left[\left(p_{R}-p_{S}\right)\min\left(\mu_{R}+\varepsilon ,Q\right)\right]. \end{array}$$

In (A.1), the first part (*p*_*S*_ − *c*)min⁡(*μ*_*RS*_ + *ε*, *Q*) is the supplier's revenue and *c*_*k*_ *Q* is supplier's prepared capacity (including the machine setup cost and production planning cost). The retailer sells the products without any investment on the products, so her expected profit can be formulated by (A.2). Because *ε* is a random variable with *τ*(*ε*) and Γ(*ε*) as its probability distribution function and cumulative distribution function, respectively, based on (A.1), we can get the supplier's optimal production quantity as follows:

(A.3) Q∗=argmax⁡Q[Π¯S(Q)]=arg{max⁡Q[(pS−c)min⁡(μRS+ε,Q)−ckQ]}=μRS+Γ−1(pS−c−ckpS−c).

Γ^−1^(*ε*) is the inverse function of Γ(*ε*), so Γ^−1^((*p*_*S*_ − *c* − *c*_*k*_)/(*p*_*S*_ − *c*)) is a constant. In (A.3), the supplier's optimal production quantity *Q** is a function of *μ*_*RS*_ and can be written as *Q**(*μ*_*RS*_). Let *A* stand for Γ^−1^((*p*_*S*_ − *c* − *c*_*k*_)/(*p*_*S*_ − *c*)). We introduce (A.3) into (A.2) and take a derivative of ${\bar{\Pi }}_{R}(\mu_{RS})$:

(A.4) $$\begin{array}{c} \frac{d\left[{\bar{\Pi }}_{R}\left(\mu_{RS}\right)\right]}{d\mu_{RS}}=\left(p_{R}-p_{S}\right)\left[1-\Gamma \left(A-\mu_{R}+\mu_{RS}\right)\right]. \end{array}$$

Because Γ(·) is the distribution of random variable *ε*, Γ(*A* − *μ*_*R*_ + *μ*_*RS*_) ≤ 1, the value of (A.4) is nonnegative. This means retailer's profit increases with her reported demand. In order to maximize her profit, retailer reports the demand as much as possible. The optimal decision on reported demand for the retailer is *μ*_*RS*_* → +*∞*. According to (A.3), the supplier's profit when he fully relies on retailer reported demand is ${\bar{\Pi }}_{S}[Q^{*}(\mu_{RS}^{*}|\mu_{RS}^{*}\to +\infty )]$. We can prove that the supplier's profit function ${\bar{\Pi }}_{S}(Q)$ is concave in input material quantity *Q* (proved in Section 4). Thus the supplier's profit can be written as

(A.5) $$\begin{array}{c} {\bar{\Pi }}_{S}\left[Q^{*}\left(\mu_{RS}^{*}\;|\;\mu_{RS}^{*}⟶+\infty \right)\right]⟶-\infty . \end{array}$$

Therefore, the supplier will not fully trust the retailer reported demand information. Without an information filter contract, the supplier will make the decision only relying on his own forecasted demand. According to (A.1) and (A.3), the supplier profit function can be written as ${\bar{\Pi }}_{S}(Q)=E_{\varepsilon }[(p_{S}-c)\min(\mu_{R}+\varepsilon ,Q)-c_{k}Q]$, and the supplier's optimal product quantity can be calculated as

(A.6) $$\begin{array}{c} Q^{*}\left(\mu_{S}\right)=\arg\underset{Q}{\max}{\bar{\Pi }}_{S}\left(Q\right)=\mu_{S}+A. \end{array}$$

Then the supplier's expected profit is

(A.7) Π¯S∗=Eε[(pR−c)min⁡(μS+ε,μS+A)−ck(μS+A)]=(pR−c−ck)(A+μS)−(pR−c)∫−∞AΓ(ε)dε.

The retailer's evaluation about supplier's forecasted demand is ${\hat{\mu }}_{S}$. Based on (A.2) and (A.6), the retailer's profit can be written as

(A.8) $$\begin{array}{c} {\bar{\Pi }}_{R}^{*}=\underset{\varepsilon }{E}\left[\left(p_{R}-p_{S}\right)\min\left(\mu_{R}+\varepsilon ,{\hat{\mu }}_{S}+A\right)\right]. \end{array}$$

Equation (A.6) means the supplier makes the decision on *Q**(*μ*_*S*_) without taking the retailer's reported demand information into consideration at the asymmetric circumstance. In this situation, the retailer's profit is shown in (A.8).
